# Variable resistance to zinc intoxication among *Streptococcus agalactiae* reveals a novel *IS*1381 insertion element within the zinc efflux transporter gene *czcD*

**DOI:** 10.3389/fimmu.2023.1174695

**Published:** 2023-05-26

**Authors:** Brian R. Varghese, Kelvin G. K. Goh, Devika Desai, Dhruba Acharya, Collin Chee, Matthew J. Sullivan, Glen C. Ulett

**Affiliations:** ^1^ School of Pharmacy and Medical Sciences, Menzies Health Institute Queensland, Griffith University, Gold Coast, QLD, Australia; ^2^ Department of Medicine, University of Alabama at Birmingham, Birmingham, AL, United States

**Keywords:** *Streptococcus agalactiae*, metal ions, metallobiology, zinc efflux, *czcD*

## Abstract

*Streptococcus agalactiae*, also known as group B Streptococcus, is an important human and animal pathogen. Zinc (Zn) is an essential trace element for normal bacterial physiology but intoxicates bacteria at high concentrations. Molecular systems for Zn detoxification exist in *S. agalactiae*, however the degree to which Zn detoxification may vary among different *S. agalactiae* isolates is not clear. We measured resistance to Zn intoxication in a diverse collection of clinical isolates of *S. agalactiae* by comparing the growth of the bacteria in defined conditions of Zn stress. We found significant differences in the ability of different *S. agalactiae* isolates to resist Zn intoxication; some strains such as *S. agalactiae* 18RS21 were able to survive and grow at 3.8-fold higher levels of Zn stress compared to other reference strains such as BM110 (6.4mM vs 1.68mM Zn as inhibitory, respectively). We performed *in silico* analysis of the available genomes of the *S. agalactiae* isolates used in this study to examine the sequence of *czcD*, which encodes an efflux protein for Zn that supports resistance in *S. agalactiae*. Interestingly, this revealed the presence of a mobile insertion sequence (IS) element, termed *IS*1381, in the 5′ region of *czcD* in *S. agalactiae* strain 834, which was hyper-resistant to Zn intoxication. Interrogating a wider collection of *S. agalactiae* genomes revealed identical placement of *IS*1381 in *czcD* in other isolates from the clonal-complex-19 (CC19) 19 lineage. Collectively, these results show a resistance spectrum among *S. agalactiae* isolates enables survival in varying degrees of Zn stress, and this phenotypic variability has implications for understanding bacterial survival in metal stress.

## Introduction

*Streptococcus agalactiae* colonizes the urogenital tract in a quarter to a third of all healthy women of child-bearing age (1). Of the approximately 30% of healthy women who are asymptomatic carriers of the bacteria, half of newborns of these mothers will be exposed to *S. agalactiae* (1, 2). Transmission of *S. agalactiae* from the mother to the neonate takes places vertically, during pregnancy or during labor (3, 4). *S. agalactiae* is a major cause of early- and late-onset disease in neonates (5), which can lead to meningitis (6, 7). *S. agalactiae* also causes various infections in adults, including skin and soft tissue infections, urinary tract infections (UTIs), pneumonia and bacteremia. The overall mortality rate of *S. agalactiae* infection in adults is estimated at 15% or more in the United States (3, 8). Thus, *S. agalactiae* is a usual asymptomatic colonizer of the female urogenital tract and causes a spectrum of disease in both newborns and adults.

There is currently no licensed vaccine for prevention of *S. agalactiae* infection, despite extensive research and development efforts over the past few decades (9, 10). Increased antibiotic usage during the past decade for infection-prevention strategies aimed at *S. agalactiae* (11) is a clinical practice associated with increasing antibiotic resistance as well as hyper-virulent strains of *S. agalactiae*, which is recognized as an emerging global threat (12–14). Thus, defining the mechanisms by which this pathogen causes disease in humans, including most notably, how *S. agalactiae* avoids the natural antimicrobial activities of the body’s defenses is of great importance. Such knowledge will ultimately deliver new opportunities to treat and prevent disease caused by this pathogen, such as by targeting newly defined virulence mechanisms or amplifying specific host defense pathways that are essential in the control of infection.

In bacteria, zinc (Zn) is an essential cofactor for metalloenzymes (15, 16) but is toxic at high concentrations, as can be encountered inside phagocytes (17, 18). In the host, bacterial pathogens internalize essential Zn (19, 20), but the host can restrict Zn availability as an antimicrobial strategy (21) and phagocytes can mobilise cellular Zn to expose internalized bacteria to higher metal concentrations that are antimicrobial (22–24). The innate immune response to *S. agalactiae*, for example, comprises a robust Zn mobilization response in macrophages (25). Host-driven Zn intoxication of bacteria may involve ablation of uptake of essential Mn (26), a compromised bacterial response to oxidative stress (27), or disrupted central carbon metabolism (28). Evidence of a role for metal intoxication of bacteria based on Zn as an antimicrobial of innate immunity has emerged only relatively recently, as reviewed elsewhere (17, 24).

To counteract actions of the innate immune response some bacteria can evade metal intoxication using mechanisms of detoxification that involve metal efflux (29). In *S. agalactiae*, a genetic system that manages Zn homeostasis by regulating metal import and export *via* a Zn-sensing transcriptional response regulator, encoded by *sczA*, and a Zn efflux transporter, encoded by *czcD*, has recently been described (25). The *czcD*-*sczA* Zn-management axis supports virulence of *S. agalactiae* by facilitating bacterial survival in the host during systemic infection (25). Zinc transport machinery in *S. agalactiae* appears to be expendable when the Zn-limiting pressure of calprotectin, a metal chelator abundant in neutrophils, is absent (30). In addition, regulatory cross-talk in *S. agalactiae* at the level of the copper-sensing transcriptional regulator CopY can support resistance to Zn intoxication (31). Knowledge of molecular management of Zn homeostasis in *S. agalactiae* is largely based on work with only a few reference strains, namely 874391 (25, 31), A909 and CJB111 (30). The degree to which different *S. agalactiae* clinical isolates may differ in sensitivity to Zn intoxication is unclear.

In this study, we assessed sixteen strains of *S. agalactiae* from diverse capsular serotypes, sequence types and isolation sources to compare relative resistance to Zn intoxication. *S. agalactiae* strains exhibited significant differences in Zn-resistance phenotypes and the implications for host-pathogen interactions are discussed.

## Materials and methods

### Bacterial strains and growth media

The bacterial strains used in this study are outlined in **Table S1**. *S. agalactiae* were routinely grown at 37°C, with agitation at 200rpm in Todd-Hewitt Broth (THB) or on TH agar (1.5% w/v). To enumerate bacteria grown in liquid cultures we performed routine retrospective colony counts by plating dilutions of bacteria on tryptone soya agar containing 5% defibrinated horse blood (Thermo Fisher Scientific). Media were supplemented with antibiotics (spectinomycin (Sp) 100μg/mL; chloramphenicol (Cm) 10 μg/mL), as indicated.

### Measurement of *S. agalactiae* growth during conditions of Zn stress

To compare the growth of different bacterial strains we performed growth assays with 200μL culture volumes in 96-well plates (Greiner) that were sealed using Breathe-Easy membranes (Sigma-Aldrich); growth was measured as attenuance (D, at 600nm) using a ClarioSTAR multimode plate reader (BMG Labtech) in Well Scan mode using a 3mm 5x5 scan matrix with 5 flashes per scan point and path length correction of 5.88mm, with agitation at 300rpm and recordings taken every 30min. Media for growth assays was THB supplemented with Zn (supplied as ZnSO_4_) as indicated. For attenuance baseline correction, D_600nm_ values were corrected against a cell-free blank of THB or THB with equivalent supplemental zinc. We also measured CFU/mL at 18h by performing retrospective colony counts to estimate the number of viable cells in each condition at the end of the assay period.

### DNA extraction, genetic modification of *S. agalactiae*, and qRT-PCR

Plasmid DNA was isolated using miniprep kits (QIAGEN), with modifications for *S. agalactiae* as described elsewhere (32). Plasmids and primers are listed in **Tables S2, S3**, respectively. An isogenic *czcD*-deficient mutant of wild-type (WT) *S. agalactiae* 834, termed 834Δ*czcD*, harboring a deletion in the gene encoding the Zn efflux transporter CzcD, was constructed by allelic exchange using pHY304aad9 to replace *czcD* with a chloramphenicol (Cm) resistance marker as described previously (33, 34), using plasmid pGU2641 (25, 33, 34). The mutant was validated by PCR using primers external to the mutation site and DNA sequencing. Complementation with full-length *czcD in trans* was achieved using the *E. coli*-streptococcal shuttle vector pDL278-derivative, pGU2699 (25, 35, 36). qRT-PCR was used to analyse the capacity of Zn stress (1mM) to induce expression of *czcD* using mid-log-phase *S. agalactiae* cultures grown for 2.5 h prior to quantification of *czcD*, exactly as previously described (25).

### Bioinformatic analyses

The *czcD* gene from strain 874391 was used in a BLAST search to identify *czcD* homologues in the genomes of reference *S. agalactiae* strains (National Center for Biotechnology Information [NCBI] database) or clinical *S. agalactiae* strains (in-house database). Nucleotide sequences and protein sequences were then aligned using ClustalW within the MEGA X tool (37) and percentage identity matrices were generated. The *czcD* locus from 834 was used to probe a database of 143 *S. agalactiae* strains available on the NCBI database to identify other strains that possess the same *I*S1381 insertion within *czcD*. Molecular serotyping and sequence typing were performed using the PubMLST database (38). Sequence comparisons were generated using the Artemis Comparison Tool (39).

### Statistical analysis

Statistical analyses were performed using GraphPad Prism 9 (GraphPad Prism Software Inc., La Jolla, California). Data shown represent at least three, independent biological repeats. Statistical significance was accepted at *p* values of ≤ 0.05.

## Results

### Variation in Zn resistance phenotypes amongst different clinical and reference *S. agalactiae* isolates

**Figure 1:**
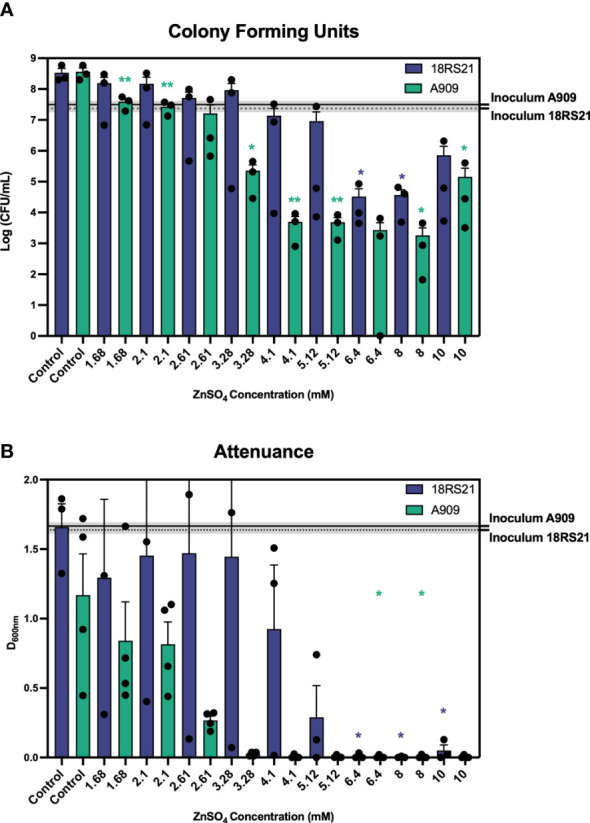
Survival of *S. agalactiae* in conditions of Zn stress showing differential resistance to Zn intoxication. *S. agalactiae* A909 and 18RS21 were grown for 18 hours prior to measuring CFU per mL **(A)** and attenuance (D_600nm_; **B**). Assays were performed in microtitre plates with starting inoculum (mean CFU/mL) represented as the black line with dashed lines and grey shading representative of the S.E.M of at least three independent experiments. Comparisons of means against the Control were performed using one-way repeated measures ANOVA; *p ≤ 0.05, **p ≤ 0.01.

We analysed the growth of 16 *S. agalactiae* strains using attenuance D_600nm_ readings to estimate bacterial population densities over time in cultures grown for 18h in media conditions of defined Zn stress. Representative strains that were i) susceptible or ii) more resistant to Zn intoxication, namely A909 and 18RS21, respectively, are shown in **Figure 1** and the remaining 14 strains are shown in **Figure S1**. The relative Zn resistance of different strains based on significant inhibitory concentration of Zn for each strain, according to relative recovery of viable cells (CFU/mL) at the end of the assay period are listed in **Supplemental Table S1**. These data show a marked variation in relative resistance to Zn intoxication among the *S. agalactiae* strains analyzed here. For example, *S. agalactiae* A909 was among the most Zn-susceptible strains; its growth was completely inhibited at ≥3.28mM Zn versus the control condition of THB without supplemental Zn [basal THB media contains 10.9 ± 0.07μM Zn (25)], according to D_600nm_ readings (**Figure 1A**) and CFU/mL estimates that showed significantly less recovery of viable A909 at ≥1.68mM Zn after 18 h of culture versus control cultures without supplemental Zn (~5x10^8^ vs ~5x10^7^, p<0.01; **Figure 1B**). Increasing the concentration of Zn >3.28mM significantly enhanced the bactericidal effect. Other strains identified to exhibit susceptibility/low relative resistance to Zn intoxication included UPSA 714, UPSA 058, ABSA 729 and BM110. The growth of these strains was significantly inhibited at between 1.68-3.28mM Zn (*p* ≤ 0.05, one-way ANOVA; **Supplementary Figure S1**), noting variability between biological quadruplicates, with significantly fewer CFU/mL recovered after 18h of culture in 1.68-3.28mM Zn (*p* ≤ 0.01, one-way ANOVA).

A high degree of relative resistance to Zn intoxication was observed in multiple strains including 18RS21 (**Figure 1**), 807, 515 and NCTC 8181 (**Supplementary Table S1**). *S. agalactiae* 18RS21 exhibited the highest level of resistance to Zn with at least 6.4 mM required to significantly inhibit growth and reduce recovery of viable cells after 18h of culture compared to control cultures without supplemental Zn (**Figure 1**; *p* ≤ 0.05, one-way ANOVA; **Supplementary Table S1**). *S. agalactiae* CJB111 was significantly inhibited for growth in 4.1mM Zn and showed a 4-log_10_ reduction in CFU/mL (*p* ≤ 0.05, one-way ANOVA) (**Supplementary Figure S1**); similarly, *S. agalactiae* 834 showed a 99.99% reduction in CFU/mL at 5.12mM Zn (*p* ≤ 0.05, one-way ANOVA). Taken together, these results show a clear separation in levels of relative resistance to Zn intoxication among different *S. agalactiae* strains; where recovery of viable cells was significantly inhibited by ≤3.28mM Zn we classified a strain as exhibiting low relative Zn resistance, similarly for >3.28mM and ≤5.12mM a strain was classified as medium resistance, and ≥6.4mM as high relative resistance; (*e.g.*, low relative resistance for A909, UPSA 714 compared to relatively resistant strains 18RS21, UPSA 807).

**Figure 2:**
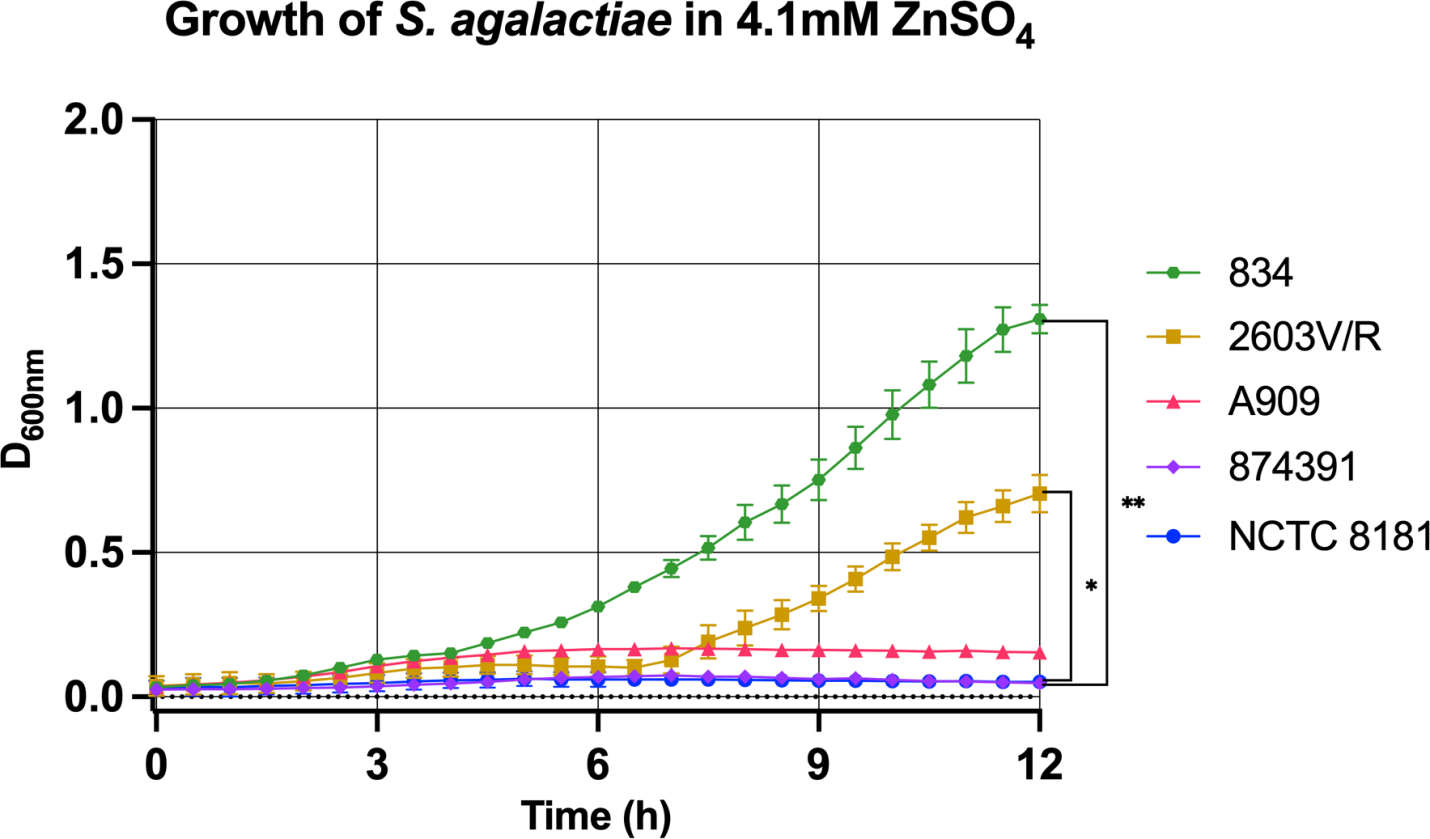
Distinct growth phenotypes of *S. agalactiae* in conditions of Zn stress. *S. agalactiae* strains, representative of distinct growth phenotypes, were grown for 12 hours in the presence of 4.1mM ZnSO_4_ with attenuance (D_600nm_) measured every 30 min and used to derive growth curves. Each data point represents an independent experiment and is shown with S.E.M. of at least n=3 experiments for each strain. Comparisons were performed using one-way repeated measures ANOVA to determine statistical significance; *p ≤ 0.05, **p ≤ 0.01.

To further characterize relative resistance to Zn stress among the *S. agalactiae* strains, we generated growth curves for a sub-selection of five strains with Zn resistance phenotypes of high and low relative resistance classified using the cut-offs described above. Comparing these strains at a midpoint concentration of Zn, 4.1mM, further highlights the distinct phenotypes of Zn resistance between these strains; strain 834 exhibits significantly more growth in this level of Zn stress compared to other strains such as 2603V/R, 874391 and NCTC8181, for example (**Figure 2**). More detailed analysis of the growth curves of individual strains across a titration of Zn concentrations demonstrated other phenoptyic distinctions; for example, analysing strain A909 showed growth without supplemental Zn with a lag-phase of 1h, followed by log-phase from 1.5h to 4h prior to slower growth up to 12h (**Supplementary Figure S2**). In contrast, cultures of A909 that contained any supplemental Zn, even the lowest concentration tested (1.68mM) were significantly inhibited for growth. The lowest amount of supplemental Zn caused a delay of the bacteria entering log-phase until 3h, that lasted until 6h prior to stationary phase until 12h. Higher amounts of Zn completely ablated the growth of A909 (*e.g.*, ≥4.1mM; **Supplementary Figure S2**). Analyzing *S. agalactiae* 834 demonstrated a log-phase from 3h to 5h followed by a decline phase up to 12h. Supplemental Zn caused incremental inhibition of growth of 834; even at the highest Zn concentration (6.4mM) growth was detectable even with an extended lag-phase of up to 6h (**Supplementary Figure S2**).

### Novel insertional element in the *czcD* region of Zn-resistant *S. agalactiae* 834

**Figure 3:**
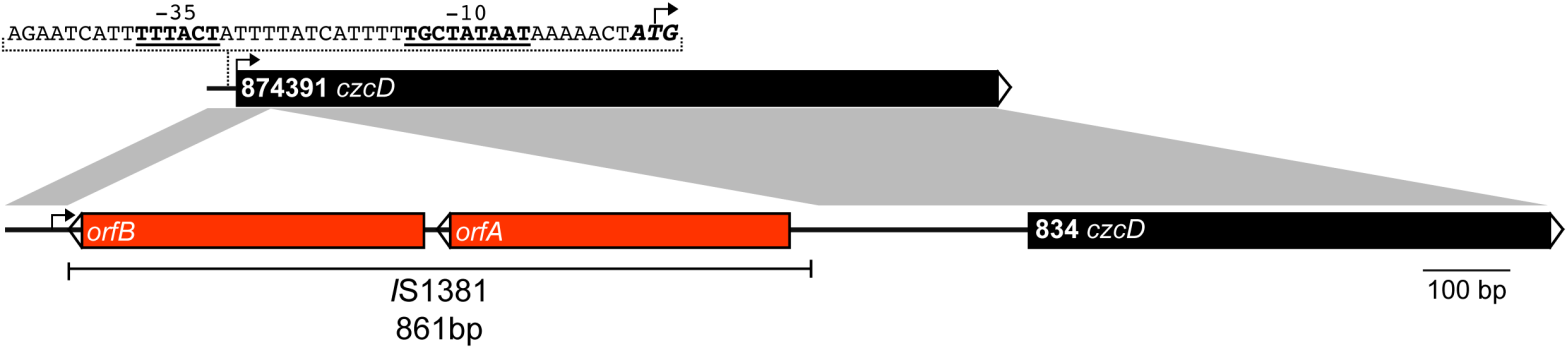
Physical map and linear comparison of *czcD* from *S. agalactiae* 874391 and 834. The black bars represent *czcD*, and the two red bars represent the *orfA* and *orfB* transposase genes within the *IS*1381 insertional element. The putative -35 and -10 transcription start sites (underlined and bold) are indicated, alongside the ATG start site of the gene (bold and italicised). The black arrow on each bar represents the ATG start site of *czcD*. The grey shaded regions represent a nucleotide identity of >99%.

Our results show that the Zn resistance phenotype varies significantly between different *S. agalactiae* strains. To investigate this further, we performed an *in silico* analysis of the *czcD* gene of the strains in this study, which have complete genomes available on the NCBI database. We found an overall high degree of sequence similarity to the genome-reference 874391 strain (**Supplemental Table S2**). Excepting single nucleotide polymorphisms (SNPs) detected in A909, 729 (position 93 A>T), 2603V/R and 1014 (position 228 T>C) and equating to 99.88% sequence identity (**Supplementary Table S2**), most other strains exhibited 100% sequence conservation in the *czcD* region. Conservation of amino acid sequence for these strains was 100% comparing to 874391 indicating redundancy in the SNPs among the strains. Strain 834 was an exception to the high degree of overall sequence similarity – this strain possessed only 49.20% identity to 874391 over the full length of the *czcD* open reading frame (**Figure 3** and **Supplementary Figure S3**). This major difference could be accounted for by the presence of an 861 bp insertion in the 5′ region of *czcD* in the *S. agalactiae* 834 genome, 34bp after the ATG start site. The 861 bp insert was identical to a known mobile genetic element termed *IS*1381, first identified in other *Streptococcus* spp. (40). The makeup of *IS*1381 in this region comprised two open reading frames (ORFs) termed *orfA* and *orfB* (**Figure 3**), each encoding putative transposase genes. Further *in silico* analysis of a collection of one-hundred and forty-three complete *S. agalactiae* genomes available on NCBI database revealed six other strains that also contained an identical *IS*1381 insertion in the 5′ region of *czcD.* Interestingly, these six strains are all serotype III and of the clonal-complex-19 (CC-19) lineage (**Supplementary Table S1**). Despite the large insertion of *IS*1381 in the *czcD* gene, *S. agalactiae* 834 exhibited a higher degree of resistance to Zn stress compared to multiple other strains, including A909, 874391, UPSA 714, UPSA 058, ABSA 729 and BM110 (**Figure 2**; **Supplementary Figure S1**, **Table S1**). Interestingly, an analysis of the draft genome of 18RS21 revealed that it also possessed the same *IS*1381 insertion within *czcD* as 834 (**Supplementary Figure S3**). 18RS21 exhibited the highest degree of resistance to Zn stress among all the strains tested in this study. Notably, however, the genomes of other strains with high Zn resistance phenotypes, including 515, UPSA 807 and NCTC 8181 were examined and were shown to not carry *IS*1381 in *czcD*.

### Mutation and analysis of the *czcD* region in *S. agalactiae* 834

**Figure 4:**
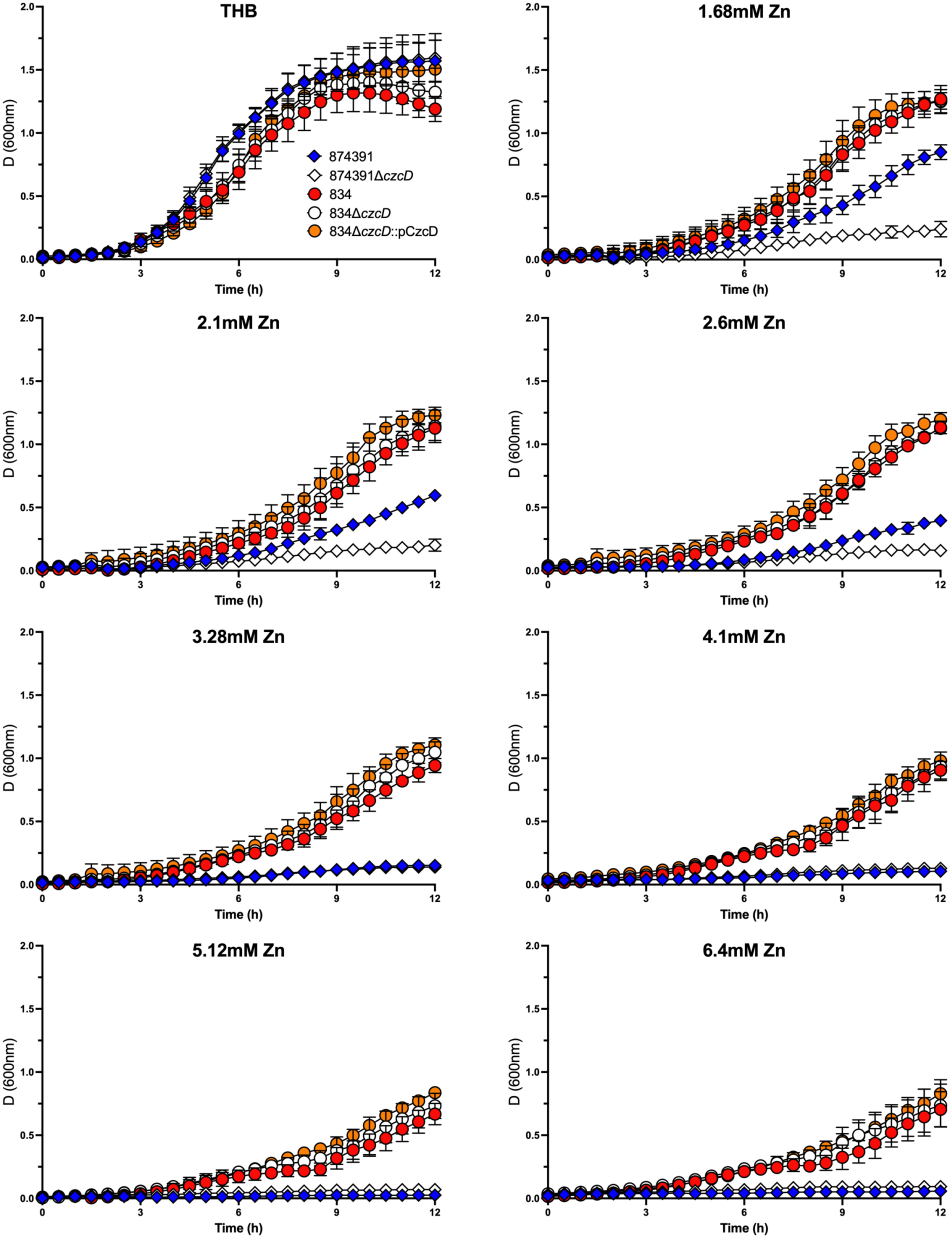
Growth kinetics of *S. agalactiae* 874391, 874391Δ*czcD*, 834, 834Δ*czcD*, and 834Δ*czcD*::PCzcD in the presence of increasing concentrations of Zn. The measurements of growth were derived from D_600nm_ readings over 12 hours, shown as mean ± SD. Data were obtained from three independent experiments.

To assess whether the *IS*1381 insertion element located in *czcD* of 834 might alter gene function in support of Zn resistance, we generated a *czcD* null mutant (*i.e.*, lacking both *IS*1381 and *czcD*) and compared the growth of this mutant to WT 834. To do this, we used our previously designed pGU2461 plasmid that was used to delete full length *czcD* in *S. agalactiae* 874391. We also generated a complemented derivative of the null mutant, 834Δ*czcD*::pCzcD into which we cloned full length *czcD* from WT 874391 *in trans*. Interestingly, analysis of the growth curves demonstrated that WT 834, 834Δ*czcD* and 834Δ*czcD*::pCzcD were each able to grow well across the range of Zn concentrations tested, including 6.4mM Zn, without significant differences in the growth of the strains in any level of Zn tested (0mM-6.4mM), notably regardless of the presence of *czcD*. Each strain grew to high culture densities even in 6.4mM Zn conditions (**Figure 4**). In comparison, WT 874391 was able to grow to high culture densities only in lower concentrations of Zn (1.68mM-2.6mM), as consistent with prior assays (**Supplementary Figure S2**). There were also no significant differences in the growth curves of the strains in control conditions of no Zn. qRT-PCR analysis of WT 834, 834Δ*czcD*::pCzcD, 874391 and 807 to compare *czcD* expression in response to Zn stress showed significant differences in the response of these strains. The lowest expression ratio was detected in strain 834 and the highest response (five times higher than that of 834) was observed in 874391 (**Supplementary Figure S4**).

## Discussion

Two recent studies established that Zn exhibits antimicrobial activity against *S. agalactiae* (25, 41). In a genomic context, the *S. agalactiae* pan-genome, that is, the core genome that is shared by all isolates, constitutes approximately 80% of one genome, and the remainder comprises strain-specific or partially shared genes (42). Therefore, we tested a diversity of *S. agalactiae* strains, across different capsular serotypes, STs and clinical backgrounds to gain insight into the potential variability of phenotypes of susceptibility to Zn intoxication among a variety of *S. agalactiae* isolates. The central finding of this study is the sixteen *S. agalactiae* strains tested, that include several well-characterised, genome-sequenced reference strains exhibit a range of distinct phenotypes of Zn resistance with strains being classified as high or low-level Zn resistant. Such differential resistance to Zn intoxication among different *S. agalactiae* strains is phenotypically apparent in growth assays where strains can grow to varying degrees, including to high culture densities in defined levels of Zn stress. Additionally, these phenotypic distinctions are evident in time-kill assays in which some strains are killed by Zn stress conditions in which other more Zn resistant strains can survive and grow. This study also identifies a novel *IS*1381 insertion element within the Zn efflux transporter gene *czcD* that in *S. agalactiae* strain 834, along with *czcD* itself, appears to be redundant for the relative high resistance to Zn intoxication in this strain. Our finding of no significant difference in the growth curve of the 834Δ*czcD* null mutant compared to either the WT 834 or the 834Δ*czcD*::pCzcD complemented strain in Zn stress is surprising, given the contribution of CzcD in support of resistance against Zn stress in GBS strain 874391 (25). These data suggest that *S. agalactiae* 834 utilizes yet-to-be-characterized mechanisms, unrelated to *czcD*, to support resistance against Zn stress.

Our analysis of *czcD* transcriptional responses to 1 mM Zn amongst different strains was striking and negatively correlated with the relative Zn resistance phenotypes observed in comparing growth of strains 874391 and 834. For example, in 834, which has a high level of relative Zn resistance, *czcD* trancription was low following exposure to 1 mM Zn. Conversely, in 874391 that was determined to have low relative Zn resistance, *czcD* transcription was high, in identical growth conditions. This may be explained by differences in intracellular Zn content in these strains that were exposed to identical extracellular Zn levels. In future work it would be of interest to directly quantify intracellular Zn across a panel of *S. agalactiae* isolates with different relative Zn phenotypes. These data also support the notion that there are mechanisms separate to those driven by *czcD* that support resistance to Zn stress. A recent report that *covR* promotes resistance to Zn intoxication, and *stp1/stk1* are essential for Zn resistance in GBS 874391 (31) highlights several genes beyond *czcD* that effect Zn detoxification. Additionally, *cadD* that mediates cadmium resistance in *S. agalactiae* also supports Zn resistance and promotes intracellular bacterial survival in macrophages and ascending infection during pregnancy (43). While we analyzed variation in *czcD* sequence for strains showing distinct phenotypes of Zn resistance in the current study, sequence analysis of other genes such as *covR/S, stp1/stk1* and *cadD* would be interesting to explore potential correlations with variable Zn resistance. Taken together, this suggests that variable resistance to Zn intoxication among different *S. agalactiae* strains can involve mechanisms mediated by elements separate to CzcD to support resistance to Zn stress.

In the innate immune response to infection phagocytes can mobilise cellular Zn to expose bacteria that are located inside the phagocyte to metal concentrations that are antimicrobial (22–24). Recently, we showed that Zn mobilization occurs in the macrophage response to *S. agalactiae* (25). Some bacteria evade metal intoxication by mechanisms that involve metal efflux (29); and for *S. agalactiae*, Zn efflux pairs the SczA Zn-sensing transcriptional response regulator with the Zn efflux transporter CzcD (25). Zn stress in *S. agalactiae* also dysregulates transcription of numerous core metabolic pathways, including several for synthesis of purines, pyrimidines, riboflavin, and deoxynucleoside triphosphates. In this context, mediators additional to the SczA-CzcD Zn management axis regulate cellular management of intracellular Zn levels in *S. agalactiae*, including *arcA*, for example, which supports resistance to Zn intoxication (25). Other changes in *S. agalactiae* that occur because of Zn stress include broad dysregulation of cellular metal management aside from Zn itself; encompassing transcriptional shifts in the activities of putative manganese (Mn), iron (Fe), and nickel (Ni) transport loci, and major changes in the cellular pools of Mn and Fe (25). Thus, the roles of these metals in *S. agalactiae* strains that exhibit distinct Zn resistance phenotypes would be of interest. In this work, we focused on degrees of variation in resistance to Zn intoxication among different *S. agalactiae* strains and, subsequently, the presence of a novel *IS*1381 insertion element within *czcD* and how it might alter function. It will be of interest in the future to investigate the role of genes that influence Zn resistance other than *czcD*, including *arcA*, and genes for Mn, Fe, Ni (25) and Cd (43) transport in *S. agalactiae* strains such as 834 (Zn resistant) and A909 (Zn sensitive) under conditions of Zn stress.

A 2022 study by Francis et al. reported antimicrobial properties of Zn against a range of *S. agalactiae* strains, and suggested that isolation source, capsular serotype, and ST might contribute to susceptibility or resistance to Zn stress (41). Comparing the findings of that study with the current work shows consistency in relation to phenotypes of strains such as *S. agalactiae* A909 and COH1, for example, that are Zn susceptible and relatively resistant, respectively. Most of the strains tested however are unique to either of the two studies, precluding direct comparisons. Interestingly, the strains more susceptible to Zn intoxication in the current study, including BM110 and 874391 were of capsular type III; Francis et al. found that capsular type III strains of ST-19 exhibited the highest resistance to Zn intoxication (41). In our study, we cannot ascribe any clear role for capsular type in the phenotype of strains for Zn resistance, noting the number of isolates analysed in the current study was half the number of the study by Francis et al., which is a limitation of the present work. Analysis of more strains will be useful to further our understanding of the range of Zn resistance phenotypes in *S. agalactiae*.

A novel finding of this work is the identification of *IS*1381 in the 5′ region of *czcD* in *S. agalactiae* strain 834 that was relatively resistant to Zn intoxication. Insertion sequences can modify phenotypes by upregulating or inactivating proximal genes (44, 45). In *S. agalactiae*, some nonhemolytic strains were found to have an *IS*1381-variant integrated into an essential component of the toxin (46). Hence, we examined how the *IS*1381 element might affect the high Zn resistance observed in 834; to investigate the hypothesis that *IS*1381 was acting to increase the resistance of *S. agalactiae* 834 to high levels of Zn, we constructed a null mutant in 834*ΔczcD* and complement strain using full length *czcD* from WT 874391. Comparing the growth of these strains under conditions of Zn stress showed intriguingly that deletion of *czcD* and overexpression of prototypical *czcD in trans* did not significantly affect the growth of 834 in different concentrations of Zn. This result is surprising because the *czcD* gene encodes for a Zn efflux protein and is required by *S. agalactiae* for resistance against Zn stress (25); therefore, the null mutant was expected to be rendered more susceptible to Zn stress. However, a recent study used a transposon-insertion screen that identified a suite of genes that *S. agalactiae* uses to overcome Zn intoxication, that are separate to *sczA*-*czcD* Zn management (31). Therefore, it would be interesting to examine the function of these genes in 834 during growth in Zn stress to examine other factors which may contribute to this strain’s relative resistance to Zn. It is also noteworthy that *IS*1381 integrates at multiple sites across a bacterial genome, at seven other loci in the instance of *S. agalactiae* strain 834 including downstream of *guaA* involved in purine biosynthesis (47). It would be of interest to examine these additional sites for their contribution to surviving Zn intoxication.

We approached the measurement of resistance to Zn stress in *S. agalactiae* by using two measurements of attenuance and recovery of viable cells in cultures grown in THB, a widely utilized medium for β-haemolytic streptococci but one that does not closely represent the natural niches that *S. agalactiae* thrives in within humans and animals. Use of THB in this study provided new insight into the diversity of Zn resistance phenotypes among a defined collection of strains, and having established variations in Zn resistance among the strains in this study it would be of interest to extend the findings to chemically defined minimal medium, such as CDM, which more closely reflects a nutrient-limited host environment. It is of note that in growth assays of *S. agalactiae* in CDM, Zn exhibited elevated toxicity, since sub-millimolar levels (0.25mM) were sufficient to significantly inhibit wild-type *S. agalactiae* 874391 (25). It is also noteworthy, then, that the assays of Francis et al. used Brain Heart Infusion broth to compare phenotypes amongst *S. agalactiae* strains. Precisely how this medium differs from THB is yet to be determined, but collectively these observations imply that the specific growth conditions under which Zn intoxication assays are performed have a marked impact on the experimental findings.

## Data availability statement

The original contributions presented in the study are included in the article/**Supplementary Material**. Further inquiries can be directed to the corresponding authors.

## Author contributions

Conceptualization: MS, GU and KG. Data curation: MS, KG, GU and BV. Formal analysis: MS, KG, GU and BV. Funding acquisition: MS, KG and GU. Investigation: MS, KG, GU, BV, DD, DA and CC. Methodology: MS, KG, GU, BV and DD. Project administration: GU and MS. Resources: GU. Supervision: GU, MS and KG. Validation: MS and KG. Writing – original draft: GU and BV. Writing – review & editing: MS, KG and GU. All authors contributed to the article and approved the submitted version.
