# Supplementary material for: Variable resistance to zinc intoxication among Streptococcus agalactiae reveals a novel IS1381 insertion element within the zinc efflux transporter gene czcD
Source: Front Immunol. 2023 May 26;14:1174695. doi: 10.3389/fimmu.2023.1174695 (PMC10251203; doi:10.3389/fimmu.2023.1174695)
Supplement: Supplementary file 1 [file DataSheet_1.pdf]

Variable resistance to zinc intoxication among *Streptococcus agalactiae* reveals a novel *IS*1381 insertion element within the zinc efflux transporter gene *czcD*

Brian R Varghese[1*], Kelvin G K Goh[1*], Devika Desai[1], Dhruba Acharya[1], Colin Chee[1], Matthew J Sullivan[1,2†], and Glen C Ulett[1,3†]

[1]School of Pharmacy and Medical Sciences, and Menzies Health Institute Queensland, Griffith University, Gold Coast Campus, QLD, Australia 4222

[2]School of Biological Sciences, University of East Anglia, Norwich, NR4 7TJ, United Kingdom

[3]Department of Medicine, University of Alabama at Birmingham, Birmingham AL 35294

Supplementary Material

Supplemental Table S1: *S. agalactiae* isolates used in this study.

Supplemental Table S2: Genome sequenced *S. agalactiae* strains that contain an identical *IS*1381 insertion in *czcD* as compared to *S. agalactiae* 834.

Supplemental Table S3. Summary of Zn resistance phenotypes among *S. agalactiae* strains, according to attenuance and cell viability measures, and ranked according to relative Zn resistance based on recovery of viable CFU/mL at end of assay period.

Supplemental Table S4. Summary of the nucleotide and amino acid identity of CzcD

from the different strains against reference *S. agalactiae* strain 874391. Only strains with complete genomes available on the NCBI database are included.

Supplemental Figure S1. Survival of *S. agalactiae* in conditions of Zn stress showing differential resistance to Zn intoxication. *S. agalactiae* 714 (A), 058 (B), NEM316 (C), 729 (D), COH1 (E), 515 (F), 2603V/R (G), NCTC 8181 (H), 807 (I), BM110 (J), 834 (K), 874391 (L), CJB111 (M) and 1014 (N) were grown for 18 hours prior to measuring attenuance (D_{600nm}; blue), and colony-forming units ((CFU) per mL; green). Assays were performed in microtitre plates with starting inoculum (mean CFU/mL) represented as the black line with dashed lines and grey shading representative of the S.E.M of at least three independent experiments. Comparisons of means against the Control were performed using one-way repeated measures ANOVA; *$p \leq 0.05$, **$p \leq 0.01$, *** $p \leq 0.001$.

Supplemental Figure S2. Distinct growth phenotypes of *S. agalactiae* in conditions of Zn stress. *S. agalactiae* strains A909 (A), 834 (B), 2603V/R (C), 874391 (D) and NCTC8181 (E) were grown for 12 hours in the presence of increasing concentrations of $ZnSO_4$ with attenuance (D_{600nm}) measured every 30 min and used to derive growth curves. Each data point represents an independent experiment and is shown with S.E.M. of at least n=3 experiments for each strain and condition. Comparisons of means against the Control (0mM) were performed using one-way repeated measures ANOVA; *$p \leq 0.05$, **$p \leq 0.01$, *** $p \leq 0.001$.

Supplemental Figure S3. Nucleotide alignment of *czcD* from strain 834 and 18RS21. Red arrows represent the *orfA* and *orfB* transposase genes, and the black

arrow represents *czcD*. Dots represent nucleotide identity between the two sequences. The first 40 bp from 18RS21 are missing and represented by a dash (-) as the sequence was extracted from the end of a contig.

Supplemental Figure S4. Expression analysis of *czcD* in response to Zn stress in *S. agalactiae* strains. Expression ratio (fold change) of *czcD* within each strain quantified by qRT-PCR in THB medium containing 1.0 mM Zn, compared to THB without Zn. Bars show mean and SD of 4 independent experiments.

Supplemental Table S1: *S. agalactiae* isolates used in this study.

Streptococcus agalactiae

Strain designation	Comments	Source
874391	ST-17(17), Serotype III, vaginal isolate	[48; 49]
NEM316 (ATCC-12403)	ST-23(23), Serotype III, neonatal sepsis	[50]
COH1 (ATCC BAA-1176)	ST-17(17), Serotype III, neonatal sepsis	[51]
A909 (ATCC BAA-1138)	ST-7(1), Serotype Ia, neonatal sepsis	[52]
BM110	ST-17(17), Serotype III, vaginal isolate	[53]
515 (ATCC BAA-1177)	ST-23(23), Serotype Ia, neonatal CSF	[54]
CJB111 (ATCC BAA-23)	ST-1(1), Serotype V, blood	[55]
2603V/R (ATCC BAA-611)	ST-110(19), Serotype V	[56]
NCTC 8181 (ATCC 13813)	ST-7, non-typeable, milk	[57]
18RS21 (ATCC BAA-1175)	ST-19(19), Serotype II	[58]
ABSA 1014	ST-28(19), Serotype II, urine	[33]
ABSA 729	ST-452(24), non-typeable, urine	[33]
ABSA 834	ST-182(19), III, urine	[33]
UPSA 807	ST-1(1), V, urine	[33]
UPSA 714	ST-17(17), III, urine	[33]
UPSA 058	ST-23(23), III, urine	[33]
GU3155	834Δ*czcD* (*czcD*$^-$ mutant); Cm	This work
GU3201	*czcD*-complement (pGU2699) in GU3155, Sp	This work
**Plasmids**		
pHY304aad9	*ori* (Ts); temperature-sensitive shuttle vector; Sp	[33]
pGU2461	pHY304*aad9*-derivative *czcD*Δ::*Cm* construct; Cm, Sp	[25]
pGU2699	*czcD* cloned into pMSP3545spec; Sp	[25]
**Primers**		
czcD-Chk-F1	TGTCTTTGATGAAGCCACCA	
czcD-Chk-R1	TCGAAAGGTGTATTTCCGATG	
Cm-Up-R1	CGTTTGTTGGTTCAAATAATGA	
Cm-Down-F1	TTCCATGGACTTCATTTACTGG	

Supplemental Table S2: Genome sequenced *S. agalactiae* strains that contain an identical *IS*1381 insertion in *czcD* as compared to *S. agalactiae* 834.

Strain name	Genbank Accession	Start	End	Capsule	ST	CC
HU-GS5823	AP018935.1	452769	451045	III	335	19
GCMC97051	AP020310.1	518091	516367	III	27	19
H002	CP011329.1	449953	448229	III	928	19
B508	CP021770.1	212679	210955	III	335	19
SG-M25	CP021867.1	516230	514506	III	19	19
S9968	CP058666.1	138929	140653	III	19	19

Supplemental Table S3. Relative Zn resistance of *S. agalactiae* strains used in this study based on significant inhibitory concentration of Zn for each bacterial isolate, according to recovery of viable cells (CFU/mL) at end of the assay.

Strain	Serotype	Sequence Type (CC)	Significant inhibitory Zn concentration based on:		Relative Zn Resistance
			Cell recovery (CFU/mL)	Attenuance (D_{600nm})	
BM110	III	ST-17	1.68mM	1.68mM	Low
A909	Ia	ST-7	1.68mM	2.61-3.28mM	Low
874391	III	ST-17	1.68mM	3.28mM	Low
UPSA 714	III	ST-17(17)	3.28mM	1.68mM	Low
UPSA 058	III	ST-23(23)	3.28mM	2.61mM	Low
ABSA 729	NT	ST-452(24)	3.28mM	3.28mM	Low
ABSA 1014	II	ST-28(19)	3.28mM	2.61mM	Low
NEM316	III	ST-23	4.1mM	4.1mM	Medium
COH1	III	ST-17	4.1mM	3.28mM	Medium
CJB111	V	ST-1	5.12mM	4.1mM	Medium
ABSA 834	III	ST-182(19)	5.12mM	ns	Medium
2603 V/R	V	ST-110	5.12mM	5.12mM	Medium
515	Ia	ST-23	6.4mM	2.61mM	High
UPSA 807	V	ST-1(1)	6.4mM	5.12mM	High
NCTC 8181	NT	ST-7	6.4mM	ns	High
18RS21	II	ST-19	6.4mM	6.4mM	High

Supplemental Table S4. Results of *in silico* analysis of *czcD* of *S. agalactiae* strains used in this study, comparing sequence similarity to reference 874391 strain.

Strain	Genome Accession Number	Gene Locus Tag	*czcd* nucleotide identity to 874391	CzcD amino acid identity to 874391
874391	CP022537	CHF17_00567	-	-
NEM316	AL766845.1	GBS0467	100	100
COH1	HG939456.1	GBSCOH1_0413	100	100
A909	CP000114.1	SAK_0514	99.88	100
BM110	LT714196.1	BQ8897_BM110_00567	100	100
515	CP051004.1	GRB95_02510	100	100
CJB111	CP063198.2	ID870_07125	100	100
2603V/R	AE009948.1	SAG0430	99.88	100
1014	N/A	GU1014_00520	99.88	100
729	N/A	*Draft assembly without tags*	99.88	100
834	N/A	GU0834_00599	49.2	-
807	N/A	GU0807_00627	100	100
714	N/A	GU0714_00583	100	100
58	N/A	GU0058_00498	100	100

Supplemental Figure S1.

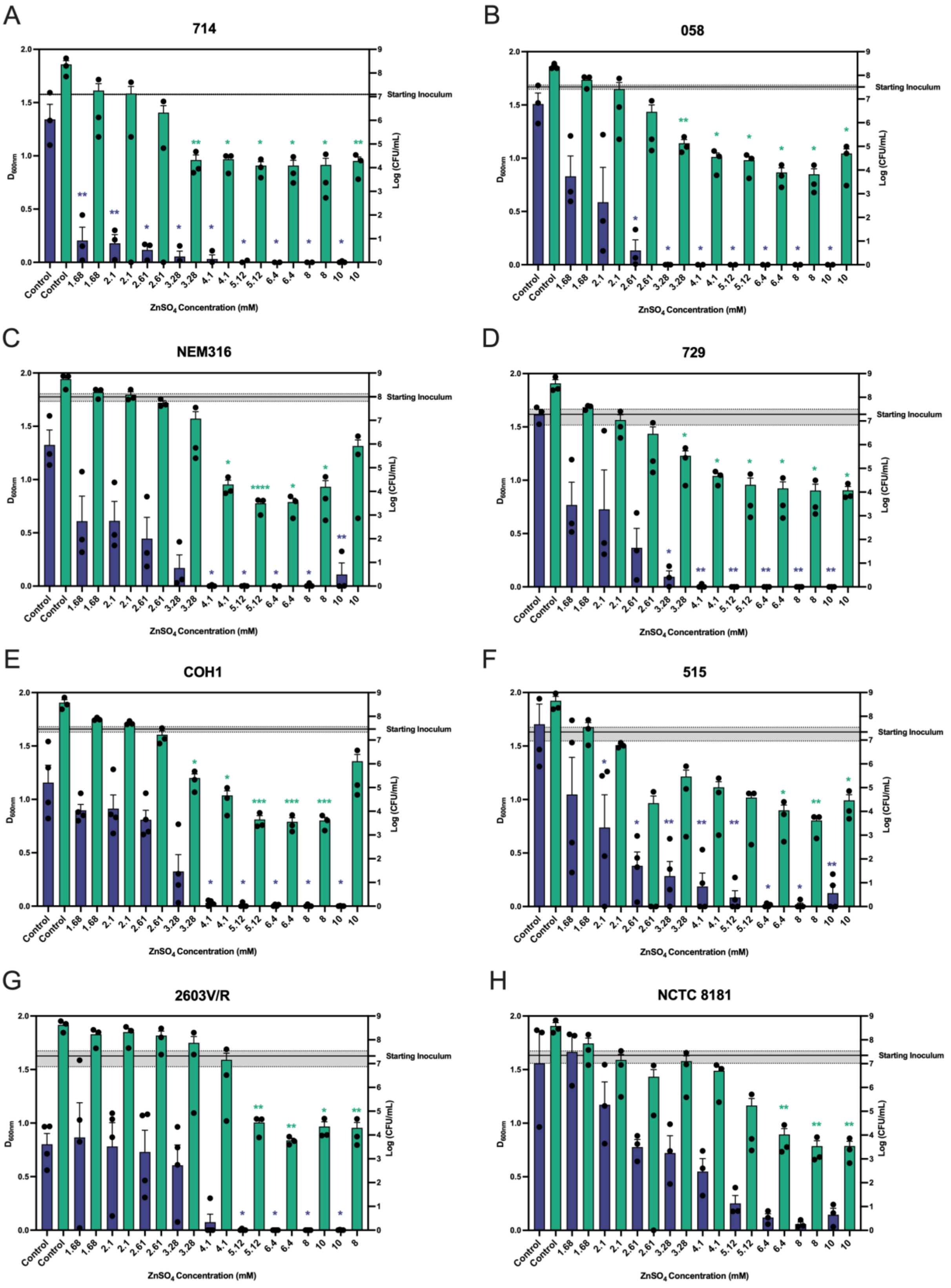

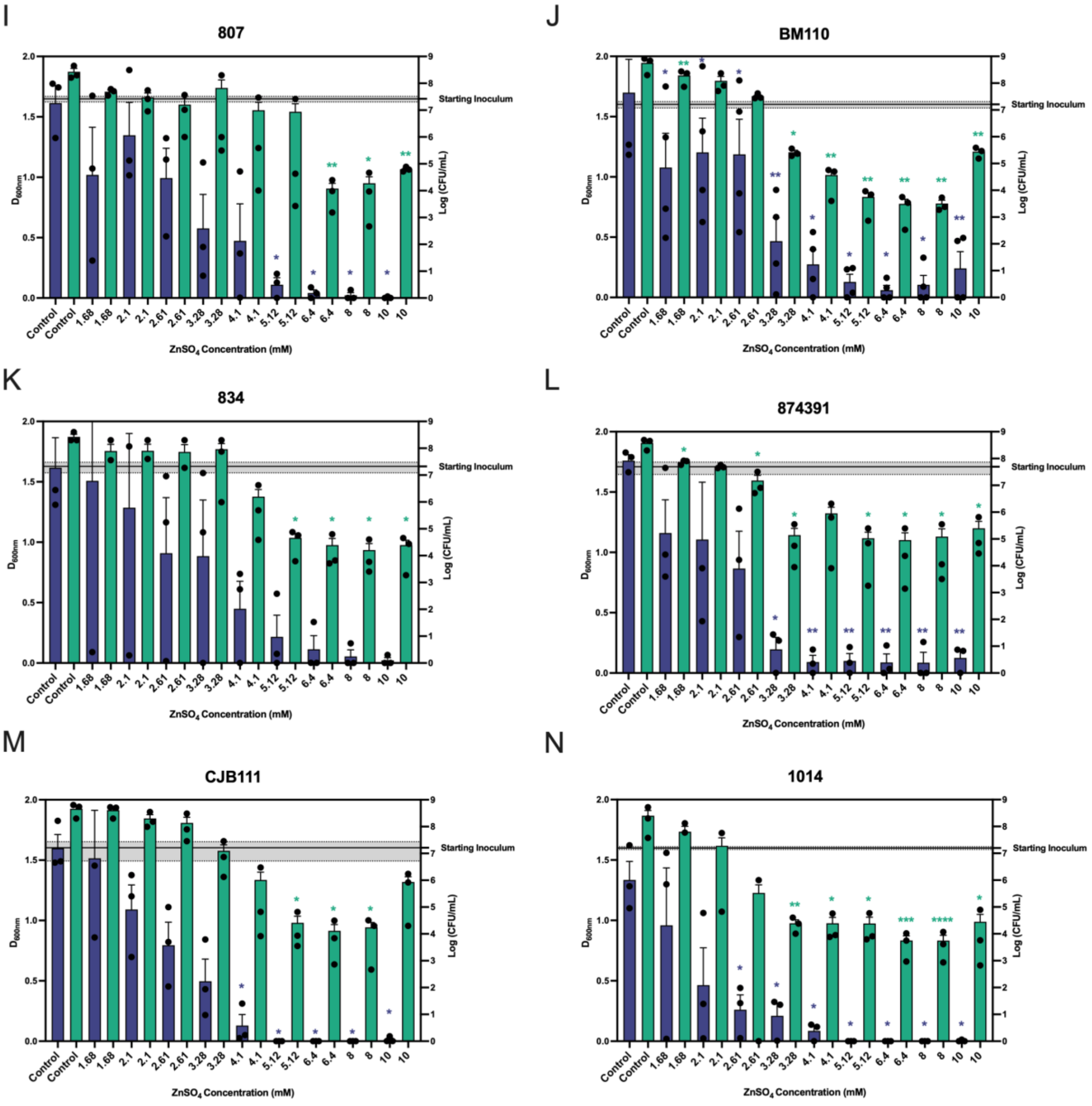
I
807
J
BM110
K
834
L
874391
M
CJB111
N
1014
Starting Inoculum
D_{600nm}
Log (CFU/mL)
Control
1.68
2.1
2.61
3.28
4.1
5.12
6.4
8
10
$ZnSO_4$ Concentration (mM)

Supplemental Figure S2.

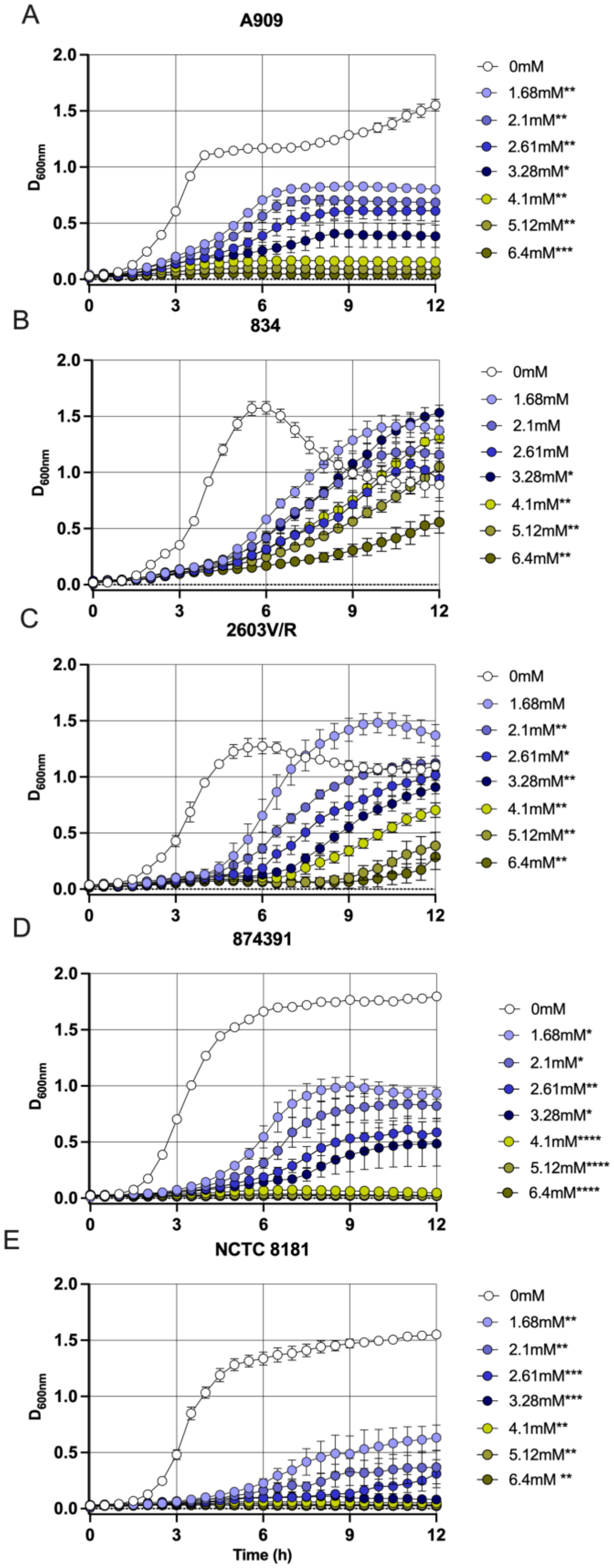

Supplemental Figure S3.

```
834czcD    ATGACATCAAACAAAAAAGTGGCTATCGCTTTTATATTAGACTTCCTGCGAAACTAAAATCCTAGTTCACG 71
18RS21czcD ----------------------------------------............................... 31
834czcD    GTTGATCATTCCAGCAATCAAATTCATTCGTAATCCAAACCGTTTGCGTCGATTTCGATAGGTTGTTGAAA 142
18RS21czcD ....................................................................... 102
834czcD    ATATTTTAAACGTTTTTACTTTGGCAAAAATATTCTCAACCTTGATTCTCTCTTTGGATAGCGTATGGTTA 213
18RS21czcD ....................................................................... 173
834czcD    TAGGTTTTATCTTCAAGAGTTAGTGGCTTAAGTTTGCTTGATTTCCTCGGAGTTTGCGCTTGTGAATACAT 284
18RS21czcD ....................................................................... 244
834czcD    CTTCATGATCCCTTGATAACCACTGTCTGCCAAGATTTTAGCAGCTTGTCCGATGTTTCTGCGACTCATTT 355
18RS21czcD ....................................................................... 315
834czcD    TGAACAACTTCATATCGTGGCAATAGTTCACTGCAATATCCAAAGAAACAATTCTCCCTTGGCTTGTGACA 426
18RS21czcD ....................................................................... 386
834czcD    ATCGCCTGAGCCTTCATAGCATGACATTTCTTTTTACCAGAATAATTCGCTAGTTGATTTTTTTAGGACGA 497
18RS21czcD ....................................................................... 457
834czcD    TTGATTTTTACCTCTGTTGCATCCACAATCACCGTATCCTCAGCACTAAGATGAGTTTTTGAAATCGTAAA 568
18RS21czcD ....................................................................... 528
834czcD    ACCACTTTGAATAAGAGTTGATTCAACCCATTGACTTCGACGGATTAAGTTGCTTTCGTGAATGCCAAAAT 639
18RS21czcD ....................................................................... 599
834czcD    CAGCCGCAATTTGTTCATAAGTGCGGTATTCTCGCATGTATTGAAGAGTAGCCATGAGGAGATCTTCTAAG 710
18RS21czcD ....................................................................... 670
834czcD    CTTAACTTAGGGGTTCGGCCACCTTTTGCGTGTTTACGTTGATAAGCTGTTTTTAACACAGCTAACATCTC 781
18RS21czcD ....................................................................... 741
834czcD    TTCAAAAGTAGTGCGCTGAACACCAACAAGGCGCTTAAATCGTACATCAGTTAATTGTTTGCTTGCTTCAT 852
18RS21czcD ....................................................................... 812
834czcD    AATTCATAGAACTATTGTACCATATTTTGTTTCGCAGGAAGTCTATTAAATATCAGCTTTTCCGTCCTTGA 923
18RS21czcD ....................................................................... 883
834czcD    ATTCATCTTTGGAAGTCTATTCTTCTCAGGAGCTATCTTAGCCGATGCTGTTCATGATTTTGGTGATGCAA 994
18RS21czcD ....................................................................... 954
834czcD    TAGCTATTGGCATCTCAGCTACTTTAGAAAAAAAAATCTAAGAAAGATGAAGATACGATATTTTCATTAGGT 1065
18RS21czcD ....................................................................... 1025
834czcD    TATAAACGTTTTAGCCTTTTAGGAGCTCTTATCACAAGTCTAATTCTTATAAGTGGATCTATCTTAGTCAT 1136
18RS21czcD ....................................................................... 1096
834czcD    GATAGAAAATATCCCAAAATTGTGGCATCCTACTCCTGTTAATTATCACGGCATGTTTATTCTAGCTGTCA 1207
18RS21czcD ....................................................................... 1167
834czcD    TCGCTATTATCATTAATGGTCTAGCTAGTTTTATTCTCCATTCTGGTCAAAGTAAACATGAGGAAATATTA 1278
18RS21czcD ....................................................................... 1238
834czcD    AGCCTTCATTTTTTGGAAGATATTCTAGGGTGGTTAGCTATTATTGTTATATCTCTTATCTTAAATTGGAA 1349
18RS21czcD ....................................................................... 1309
834czcD    ACCGTTGTATATCCTTGATCCATTATTATCAGTTGCCATATCTACTTTCATCTTATCTAAAGCACTTCCTA 1420
18RS21czcD ....................................................................... 1380
834czcD    AATTACTATCAACCCTGAAACTTTTTTTAGATGGTGTTCCAGATTCCATAGATTACGCTGCATTACATGAT 1491
18RS21czcD ....................................................................... 1451
834czcD    GAACTTAAGGGACTTTCGCAAGTAAGATCTATCAATCAACTCAATATCTGGTCAATGGATGGTATTGATAA 1562
18RS21czcD ....................................................................... 1522
834czcD    TCGAGCAATCATTCACTGTTGCTTAAATCAATTGATATCTGAAAAAGATTGTAAAAGAGCTATTCGAACGA 1633
18RS21czcD ....................................................................... 1593
834czcD    TTTGCCAACATTATAAGATAAACGATGTTACTGTTGAAATCGATTATTCTTTAAGAGAACATCAAAATCAT 1704
18RS21czcD ....................................................................... 1664
834czcD    TGCAAACCATTAAAAAACTAA 1725
           .....................
```

Supplemental Figure S4.

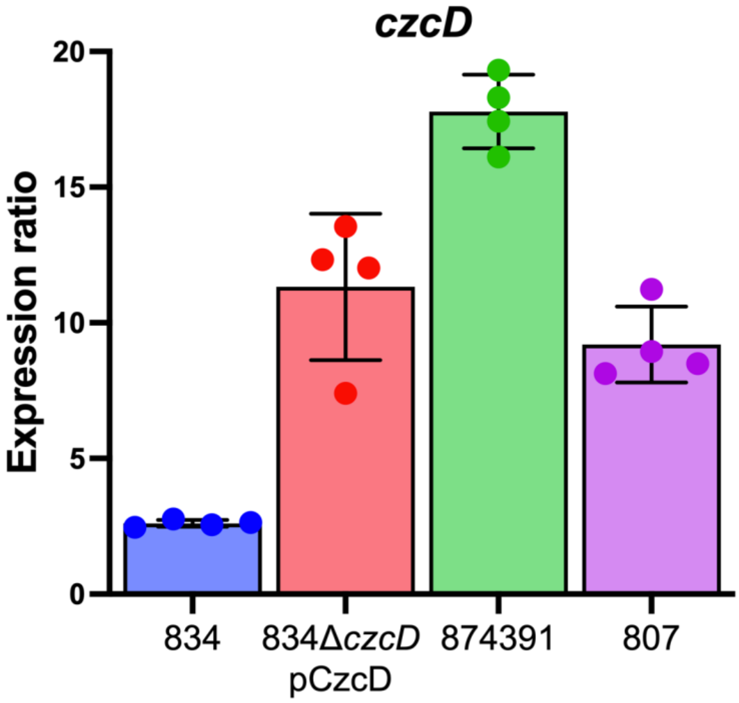

References

[48] S. Takahashi, Y. Nagano, N. Nagano, O. Hayashi, F. Taguchi, and Y. Okuwaki, Role of C5a-ase in group B streptococcal resistance to opsonophagocytic killing. Infect. Immun. 63 (1995) 4764-9.

[49] M.J. Sullivan, B.M. Forde, D.W. Prince, D.S. Ipe, N.L. Ben Zakour, M.R. Davies, G. Dougan, S.A. Beatson, and G.C. Ulett, Complete Genome Sequence of Serotype III *Streptococcus agalactiae* Sequence Type 17 Strain 874391. Genome Announcements 5 (2017).

[50] P. Glaser, C. Rusniok, C. Buchrieser, F. Chevalier, L. Frangeul, T. Msadek, M. Zouine, E. Couve, L. Lalioui, C. Poyart, P. Trieu-Cuot, and F. Kunst, Genome sequence of *Streptococcus agalactiae*, a pathogen causing invasive neonatal disease. Mol. Microbiol. 45 (2002) 1499-513.

[51] T.R. Martin, C.E. Rubens, and C.B. Wilson, Lung antibacterial defense mechanisms in infant and adult rats: implications for the pathogenesis of group B streptococcal infections in the neonatal lung. J. Infect. Dis. 157 (1988) 91-100.

[52] L.C. Madoff, J.L. Michel, and D.L. Kasper, A monoclonal antibody identifies a protective C-protein alpha-antigen epitope in group B streptococci. Infect. Immun. 59 (1991) 204-10.

[53] J.M. Musser, S.J. Mattingly, R. Quentin, A. Goudeau, and R.K. Selander, Identification of a high-virulence clone of type III *Streptococcus agalactiae* (group B Streptococcus) causing invasive neonatal disease. Proc. Natl. Acad. Sci. U. S. A. 86 (1989) 4731-5.

[54] C.J. Baker, M.S. Edwards, B.J. Webb, and D.L. Kasper, Antibody-independent classical pathway-mediated opsonophagocytosis of type Ia, group B streptococcus. J. Clin. Invest. 69 (1982) 394-404.

[55] B.L. Spencer, A. Chatterjee, B.A. Duerkop, C.J. Baker, and K.S. Doran, Complete Genome Sequence of Neonatal Clinical Group B Streptococcal Isolate CJB111. Microbiol Resour Announc 10 (2021).

[56] I.S. Aaberge, J. Eng, G. Lermark, and M. Lovik, Virulence of Streptococcus pneumoniae in mice: a standardized method for preparation and frozen storage of the experimental bacterial inoculum. Microb. Pathog. 18 (1995) 141-52.

[57] J. Ruhlmann, B. Wittmann-Liebold, D. Jurgens, and F.J. Fehrenbach, Complete amino acid sequence of protein B. FEBS Lett. 235 (1988) 262-6.

[58] R.C. Lancefield, and E.H. Freimer, Type-specific polysaccharide antigens of group B streptococci. Journal of Hygeine (Lond) 64 (1966) 191-203.
